# Unraveling the interplay between dyskinesia and overactive bladder symptoms in Parkinson’s disease: a comprehensive cohort study based on the long-term follow-up database of Parkinson’s disease

**DOI:** 10.1186/s12883-024-03578-3

**Published:** 2024-03-12

**Authors:** Hong Jin, Yiheng Du, Jiahui Yan, Jinru Zhang, Xiaoyu Cheng, Chengjie Mao, Jing Chen, Chun-feng Liu

**Affiliations:** 1 Department of Neurology and Clinical Research Center of Neurological Disease, The Second Affiliated Hospital of Soochow University, Suzhou, 215004 Jiangsu China; 2 Department of Urology, Suzhou Kowloon Hospital, Shanghai Jiaotong University School of Medicine, Suzhou, 215028 Jiangsu China; 3 Department of Urology, The Second Affiliated Hospital of Soochow University, Suzhou, 215004 Jiangsu China; 4 Jiangsu Key Laboratory of Neuropsychiatric Diseases and Institute of Neuroscience, Soochow University, Suzhou, 215123 Jiangsu China

**Keywords:** Parkinson's disease, Dyskinesia, Overactive bladder, Clinical characteristics

## Abstract

**Objectives:**

Overactive bladder (OAB) and dyskinesia are frequent complications in patients with Parkinson’s disease (PD). However, the correlation between OAB and dyskinesia has been insufficiently explored. The purpose of this study was to examine the relationship between dyskinesia, OAB, and clinical characteristics among individuals with PD.

**Methods:**

1338 PD patients were included in the present study. Demographic features were compared between patients with or without dyskinesia and OAB symptoms. Logistic regression was conducted on dyskinesia to screen clinically relevant factors. Overactive Bladder Symptom Score (OABSS) was further used to stratify the association between the severity of OAB and the occurrence of dyskinesia.

**Results:**

This study indicates that both dyskinesia and OAB are significantly related to disease severity and cognitive status. PD patients with dyskinesia and OAB having higher UPDRS scores (*p* < 0.001), H-Y scores (*p* < 0.001), NMSQ (*p* < 0.001) and MoCA scores (*p* < 0.001), and lower MMSE scores (*p* < 0.001) are identified. The multivariate logistic regression confirms that disease duration (*p* = 0.041), LEDD (*p* < 0.001), UPDRSII (*p* < 0.001), MoCA (*p* = 0.024), urgency (*p* < 0.001), frequency (*p* < 0.001), and nocturia (*p* = 0.002) are independent risk factors for dyskinesia. Trend analysis indicates that the risk of dyskinesia significantly increases when patients exhibit moderate to severe OAB symptoms (OABSS > 5) (*p* < 0.001). No significant interactions were found between OABSS and age, gender, disease duration, LEDD, and NMSQ scores in different subgroups, indicating that dyskinesia is more pronounced in patients with OABSS > 5.

**Discussion:**

This study provides compelling evidence supporting the strong correlation between OAB and dyskinesia in PD patients, emphasizing the presence of shared pathogenic mechanisms between these two conditions. Our findings underscore the importance of considering both OAB and dyskinesia in the clinical management of PD, investigating the intricate connections between OAB and dyskinesia could unveil valuable insights into the complex pathophysiology of PD and potentially identify novel therapeutic targets for more effective PD treatment strategies.

## Introduction

Parkinson’s disease (PD) is a common neurodegenerative disorder and is characterized by motor and non-motor dysfunction [1], defined by the gradual and progressive loss of dopaminergic neurons in the substantia nigra that supply the striatum [2]. Dyskinesia is a common complication of long-term use of levodopa therapy for PD [3], which is up to 36% within 5 years and up to 88% within 10 years [4–6]. Dyskinesia may manifest as involuntary twisting or writhing movements, as well as chorea-like movements in some cases. The pathogenesis of dyskinesia is unknown. Theories suggest that levodopa-induced dyskinesia is associated with neurodegenerative changes in cortical and subcortical regions, including the primary motor cortex, prefrontal cortex, basal ganglia, thalamus, and cerebellum. Researches also indicate that regions connecting those cortical and subcortical regions, including cortico-basal ganglia-thalamo-cortical and cerebello-thalamo-cortical motor pathways, are implicated in the development of levodopa-induced dyskinesia [7]. Although dyskinesia is associated with the use of levodopa, studies have shown that the retention of levodopa and early initiation of levodopa therapy does not lead to differences in motor fluctuations compared to patients who undergo delayed initiation of levodopa therapy, contrarily, early initiation of levodopa has been shown to result in greater improvements in the quality of life for patients [8]. Dyskinesia is also associated with PD subtypes, a younger age of onset, especially those with Parkin mutations, and low body weight [9, 10]. Therefore, further exploration of the relevant factors and pathogenesis of dyskinesia plays a key role in deepening our understanding of PD and seeking optimal treatment methods.

Overactive Bladder (OAB) is one of the common non-motor complications of PD [11, 12]. The most common symptom of OAB in PD is nocturia, followed by frequency and urgency [13]. Research has shown that the prevalence of OAB in PD patients varies from 27–85% [14], and PD patients have twice the risk of developing OAB compared to the general population [15]. The pathophysiology of OAB in PD is not entirely understood, but it has been linked to various factors, including abnormal bladder contractions, incomplete bladder emptying, and motor fluctuations.

There is emerging evidence to suggest a potential relationship between dyskinesia and urinary dysfunction in PD. Dopamine, the primary neurotransmitter involved in the pathophysiology of dyskinesia, also plays a critical role in the central nervous system control of bladder reflexes. Studies have shown that dopamine release due to levodopa therapy is a double-edge sword to bladder function [16]. Based on the potential shared mechanisms and the impact of dyskinesia and OAB on patients’ quality of life, we included 1,338 PD patients to investigate the relationship between OAB and dyskinesia. Our findings revealed that patient with moderate or severe OAB symptoms significantly elevated the risk of the occurrence of dyskinesia, emphasizing the systematic management of symptoms among different systems in PD treatment, in order to achieve personalized and optimal treatment outcomes. Further exploration of dyskinesia and OAB in PD patients contributes to a better understanding of the pathogenesis of PD and the identification of possible therapeutic targets.

## Materials and methods

### Raw data acquisition and patients’ informed consents

Information about patients was obtained from the Long-term follow-up database of Parkinson’s disease in Suzhou (LEAD-PD), which has been in existence since 2007. This study was approved by the Ethics Committee of the Second Affiliated Hospital of Soochow University. A total of 1338 Han Chinese PD patients from mainland China were enrolled (788 males, 58.89% of all participants), and all patients provided informed consent prior to participating in the follow-up procedure.

### Inclusion criteria

Patients were diagnosed according to the current clinical diagnostic criteria of the Movement Disorders Society (MDS) by at least two experienced neurologists [17]. These patients didn’t been given advance therapy, Such as Deep Brain Stimulation (DBS) and Levodopa/Carbidopa Intestinal Gel (LCIG).

### Exclusion criteria

The exclusion criteria for this study are as follows: (a) Patients with lower urinary tract symptoms caused by urinary tract infection, prostate enlargement, or urolithiasis who are currently receiving treatment (such as medication or surgery) or intervention measures; (b) Individuals who are unable to self-assess their urinary symptoms or complete relevant assessment questionnaires; (c) Individuals with other neurological diseases such as multiple system atrophy; (d) Patients with intellectual disabilities or who are unable to understand the research process; (e) Patients with coexisting systemic diseases such as malignant tumors, heart disease, or diabetes.

### Definition of OAB symptoms involved in this study

Urinary frequency is defined as a patient urinate more than eight times in the daytime period. Nocturia refers to the frequent need to urinate at night, usually more than twice between the time of going to bed and waking up in the morning. Urinary urgency is the term used to describe the sudden and uncontrollable need to urinate as soon as the sensation is felt. Patient experiences urinary urgency more than once a week could diagnose as urgency. Overactive Bladder Symptom Score (OABSS) is a tool that is used to diagnose OAB based on the severity of symptoms. It consists of four questions related to urinary urgency (0–5 points), urinary frequency (0–2 points), urinary incontinence (0–5 points) and nocturia (0–3 points). To calculate the OABSS score, the scores for each question are added together to give a total score which ranges from 0 to 15. Based on the OABSS score, the severity of OAB can be classified as follows: Without OAB: total score of 0–2, Mild OAB: total score of 3–5, Moderate OAB: total score of 6–11, Severe OAB: total score of 12-15 [18].

### Demographics and detailed clinical history

Demographic and clinical data included gender, age at the time of the study, disease duration, and levodopa equivalent daily dose (LEDD), Unified Parkinson’s disease rating scale (UPDRS) and Hoehn & Yahr stage (H-Y stage) scale were applied to all PD subjects during the medication “on” state [19]. Dyskinesia was assessed using UPDRS Part IV based on item 32. Item 32: “What proportion of the waking day is dyskinesia present? (Historical information)”, where a score of 0 = none, 1 = 1–25%, 2 = 26–50%, 3 = 51–75%, and 4 = 76–100%. Dyskinesia was defined as present if the response to UPDRS IV item 32 was ≥ 1. The Mini Mental State Examination (MMSE) and Montreal Cognitive Assessment (MoCA) were used to evaluate cognitive function in all participants [20]. Nonmotor symptoms were assessed using the Non-Motor Symptoms Questionnaire (NMSQ) [21].

### Statistical analysis

R software (version 4.2.0) was utilized to analyze the data. The results are presented as median (lower quartile, upper quartile). To test for statistically significant differences between two independent groups, student-t (Normal distribution) and Wilcoxson (Abnormal distribution) tests were performed. The Chi-square test was applied to compare the ratios between different groups. Univariate logistic regression and least absolute shrinkage and selection operator (LASSO) regression were used to select variations for multivariate logistic regression. Parameters with a p-value less than 0.1 and LASSO coefficient not equal to zero were selected for further multivariate analysis. A two-sided p-value of less than 0.05 was considered statistically significant. We conducted a trend analysis to assess the linear trend between the levels of OABSS and the occurrence of dyskinesia in PD patients. Interaction effects were addressed by p for interaction. A P-value less than 0.05 was considered statistically significant.

## Results

### Baseline characteristics of participants according to dyskinesia and OAB

In this study, a total of 1338 patients diagnosed with PD were included. Among them, 788 (58.89%) were male, with a median age of 64 years (interquartile range: 57 to 70 years), a median disease duration of 36 months (interquartile range: 22 to 69 months), and a median onset age of 60 years (interquartile range: 53 to 66 years). Among all patients, the incidence of urinary frequency was 24.74%, urgency was 30.49%, nocturia was 40.36%, and the incidence of dyskinesia was 22.27%. Comparing patients with or without dyskinesia and OAB symptoms, it was observed that patients with dyskinesia had significantly higher disease duration (*p* < 0.001), LEDD (*p* < 0.001), UPDRS score (*p* < 0.001), H-Y and NMSQ scores (*p* < 0.001). They also had a higher frequency of urinary frequency (*p* < 0.001), urgency (*p* < 0.001), and nocturia (*p* < 0.001), along with a higher MMSE score (*p* < 0.001) and younger onset age (*p* < 0.05) compared to patients without dyskinesia. Among patients with OAB symptoms of urinary frequency, urgency, or nocturia, LEDD (*p* < 0.001), UPDRS scores (*p* < 0.001), HY score (*p* < 0.001), incidence of dyskinesia (*p* < 0.001), and MMSE score (*p* < 0.001) were significantly higher than in patients without OAB symptoms. Furthermore, patients with nocturia and urinary frequency were older (*p* < 0.001) than those without these OAB symptoms, while the MoCA score showed no significant difference between patients with and without all three OAB symptoms. These findings indicate that dyskinesia and OAB symptoms significantly reflect the severity of PD patients’ condition and cognitive status (Table 1).

**Table 1:** Baseline characteristics of participants according to dyskinesia and OAB

Characteristics	Total	Dyskinesia		Urgency		Nocturia		Frequency	
		N	Y^sig(N vs. Y)^	N	Y^sig(N vs. Y)^	N	Y^sig(N vs. Y)^	N	Y^sig(N vs. Y)^
Gender (male%)	788(58.89%)	617(46.11%)	171(12.78%)^ns^	561(41.93%)	227(16.97%)^ns^	482(36.02%)	306(22.87%)^ns^	598(44.69%)	190(14.20%)^ns^
Age	64(57,70)	63(56,70)	65(60,70) *	63(56,70)	65(58,71)^ns^	62(55,69)	65(59,71)***	63(56,70)	66(60,71) ***
Duration	36(22,69)	36(17,60)	67(36,108)***	36(18,60)	48(24,84)***	36(17,60)	48(24,84)***	36(18,60)	56(24,96)***
Onset	60(53,66)	60(53,67)	59(51,65)*	60(53,66)	60(52,66)^ns^	59(52,66)	60(54,66.25)^ns^	59(53,66)	61(53.5,66)^ns^
LEDD	337.5(200,525)	300(200,450)	525(306,775)***	300(200,475)	400(300,650)***	300(187.5,450)	400(300,600)***	300(200,450)	450(300,675)***
UPDRS I	3(1,4)	2(1,4)	3(2,5)***	2(1,4)	3(2,5)***	2(1,4)	3(2,5)***	2(1,4)	3(2,5)***
UPDRS II	10(7,14)	9(6,12)	14(10,18.75)***	9(6,13)	11(8,16)***	9(6,12)	11(8,16)***	9(6,13)	12(8,18)***
UPDRS III	23(15,33)	21(13,29.25)	32(23,39.75)***	21(14,30)	28(19,37)***	21(13,29)	28(19,36)***	21(14,30)	29(19.5,37.5)***
UPDRS IV	1(0,3)	0(0,2)	5(4,6)***	1(0,2)	3(1,5)***	1(0,2)	2(0,5)***	1(0,2)	4(1,5)***
H-Y	2(1.5,2.5)	2(1.5,2.5)	2.5(2,3)***	2(1.5,2.5)	2.5(2,3)***	2(1.5,2.5)	2.5(2,3)***	2(1.5,2.5)	2.5(2,3)***
Frequency	331(24.74%)	154(11.51%)	177(13.23%)***	130(9.72%)	201(15.02%)***	110(8.22%)	221(16.52%)***	-	-
Urgency	408(30.49%)	222(16.59%)	186(13.90%)***	-	-	124(9.27%)	284(21.23%)***	207(15.47%)	201(15.02%)***
Nocturia	540(40.36%)	337(25.19%)	203(15.17%)***	256(19.13%)	284(21.23%)***	-	-	319(23.84%)	221(16.52%)***
Dyskinesia	298(22.27%)	-	-	112(8.37%)	186(13.90%)***	95(7.10%)	203(15.17%)***	121(9.04%)	177(13.23%)***
NMSQ	7(5,9)	7(4,8)	8(7,13) ^***^	7(4,7)	9(7,13)^***^	6(3,7)	8(7,13) ^***^	7(4,7)	10(7,14) ^***^
MMSE	27(24,29)	27(24,29)	26(22,28)***	27(24,29)	26(23,28)**	27(24.25,29)	26(23,28)***	27(24,29)	26(23,28)***
MoCA	23(20,25)	23(20,25)	23(20,25)^ns^	23(19,25)	23(20,25)^ns^	23(20,25)	23(20,26) ^ns^	23(19,25)	23(21,25) ^ns^

*Abbreviations*: *LEDD L*evodopa equivalent daily dose, *UPDRS I-III *Unified Parkinson’s Disease Rating Scale I-III, H-Y: Hoehn-Yahr Scale, *MMSE *The Mini Mental State Examination, *MoCA *Montreal Cognitive Assessment, *NMSQ *None Motor Symptom Questionnaire, *sig *significance, ns: not significant, ***p < 0.001, **p < 0.01,*p < 0.05

### Logistic regression on dyskinesia in PD patients

To further discuss the relationship between dyskinesia and OAB, we performed logistic regression on dyskinesia including gender, age, disease duration, onset age, LEDD, UPDRS I-III scores, H-Y score, MMSE, MoCA, urgency, nocturia and frequency. We excluded the UPDRS IV for predicting dyskinesia for its absolute involvement in assessing dyskinesia. In the univariate logistic regression, significant predictors (*p* < 0.1) of dyskinesia were age (*p* = 0.031), duration (*p* < 0.001), onset (*p* = 0.014), LEDD (*p* < 0.001), UPDRS I (*p* < 0.001), UPDRS II (*p* < 0.001), UPDRS III (*p* < 0.001), H-Y (*p* < 0.001), MMSE (*p* < 0.001), MoCA (*p* = 0.056), NMSQ (*p* < 0.001), urgency (*p* < 0.001), frequency (*p* < 0.001) and nocturia (*p* < 0.001). Owing to the significant collinearity of these factors, we further employed LASSO regression to screen the variables included in the multifactorial logistic regression. LASSO regression analysis found that the coefficients for age, gender, HY, and NMSQ were zero. Therefore, we included variables in the multifactorial regression analysis that satisfied both a univariate logistic regression *p* < 0.1 and LASSO regression coefficient not equal to zero. The results showed that disease duration (*p* = 0.041, OR = 1.004, 95%CI 1.000-1.008), LEDD (*p* < 0.001, OR = 1.002, 95%CI 1.002–1.003), UPDRS II (*p* < 0.001, OR = 1.106, 95%CI 1.066–1.147), MoCA (*p* = 0.024, OR = 0.975, 95%CI 0.954–0.997), frequency (*p* < 0.001, OR = 2.763, 95%CI 1.937–3.942), urgency (*p* < 0.001, OR = 3.959, 95%CI 2.780–5.636), and nocturia (*p* = 0.002, OR = 1.767, 95%CI 1.241–2.514) were independent risk factors for the occurrence of dyskinesia (Table 2).

**Table 2:** Logistic regression for clinical characteristics on dyskinesia

Characteristics	Univariate Logistic Regression		Lasso regression	Multivariate Logistic Regression	
	* p*	OR(95%CI)	Coefficient	* p*	OR(95%CI)
Gender	0.548	1.083(0.834,1.405)	0		
Age	0.031	1.015(1.001,1.028)	0		
Duration	*p* < 0.001	1.018(1.015,1.021)	0.004	**0.041**	**1.004 (1.000,1.008)**
Onset	0.014	0.984(0.971,0.997)	-0.010	0.087	0.985 (0.967,1.002)
LEDD	*p* < 0.001	1.003(1.003,1.004)	0.002	*p* < 0.001	**1.002(1.002,1.003)**
UPDRS I	*p* < 0.001	1.252(1.177,1.333)	-0.025	0.102	0.929 (0.850,1.015)
UPDRS II	*p* < 0.001	1.153(1.127,1.180)	0.089	*p* < 0.001	**1.106 (1.066,1.147)**
UPDRS III	*p* < 0.001	1.061(1.050,1.073)	0.012	0.092	1.016 (0.997,1.035)
H-Y	*p* < 0.001	2.847(2.374,3.441)	0		
MMSE	*p* < 0.001	0.938(0.912,0.964)	-0.004	0.484	0.986 (0.947,1.026)
MoCA	0.056	1.014(0.999,1.029)	-0.016	**0.024**	**0.975 (0.954,0.997)**
NMSQ	*p* < 0.001	1.193(1.160,1.229)	0		
Urgency	*p* < 0.001	6.119(4.646,8.095)	0.926	*p* < 0.001	**2.763 (1.937,3.942)**
Frequency	*p* < 0.001	8.416(6.326,11.248)	1.292	*p* < 0.001	**3.959 (2.780,5.636)**
Nocturia	*p* < 0.001	4.458(3.392,5.894)	0.489	**0.002**	**1.766 (1.241,2.514)**

Abbreviations: *LEDD *Levodopa equivalent daily dose, *UPDRS I-III *Unified Parkinson’s Disease Rating Scale I-III, *H-Y *Hoehn-Yahr Scale, *MMSE *The Mini Mental State Examination, *MoCA *Montreal Cognitive Assessment, *NMSQ *None Motor Symptom Questionnaire

### Increase in OABSS elevated the risk of dyskinesia occurrence in PD patients

To better stratify the association between OAB and dyskinesia, we divided patients into four groups based on severity of OAB symptoms. In the basic model, only OABSS was included. Logistic regression analysis revealed that when the OABSS score was generally low (OABSS range from 3 to 5), there was no statistically significant risk of dyskinesia in PD patients. However, as the OABSS score increased (OABSS range from 6 to 15), the risk of dyskinesia in PD patients significantly increased (OR: 8.251, 95% CI: 5.912,11.700), with an overall P-trend < 0.001. Subsequently, we constructed model 2 by including additional factors including onset age, duration and LEDD. Further, in model 3, we included UPDRS I, II and III scores, MMSE, MoCA and NMSQ scores. Logistic regression analysis in both models yielded similar results, indicating a significant increase in the risk of dyskinesia in PD patients with an OABSS score of 6 or higher (model 2: OR 6.028, 95% CI: 4.219,8.739, p-trend < 0.001; model 3: OR 4.376, 95% CI: 2.772–6.989, p-trend < 0.001) (Table 3). These findings revealed that a higher OABSS score, particularly when it reached 6 or above, was significantly associated with an increased risk of dyskinesia in PD patients.

**Table 3:** Association between OABSS and dyskinesia in PD patients

	Over-Active Bladder Syndrome Score (OABSS)			P trend	Per 1 SD
Dyskinesia vs. Non-dyskinesia					
OABSS interval	0–2	3–5	6–15		
Median OABSS	2	5	10		
Dyskinesia Events, n (%)	51(9.68)	26 (7.65)	221(46.92)		
Model 1	1	0.773(0.466,1.254)^ns^	8.251(5.912,11.700)^***^	<0.001	3.857(3.313,4.522)
Model 2	1	0.678(0.399,1.126)^ns^	6.028(4.219,8.739)^***^	<0.001	3.522(2.987,4.185)
Model 3	1	0.561(0.317,0.971)^*^	4.376(2.772,6.989)^***^	<0.001	3.838(3.090,4.816)

Model 1: unadjusted model (Dyskinesia vs. non-dyskinesia ~ OABSS). Model 2: adjusted for Onset, Duration, LEDD. Model 3: further adjusted for UPDRSI, UPDRSII, UPDRSIII, MMSE, MoCA, NMSQ. *Abbreviations*: *SD *Standard deviation, *Per 1 SD *Per One Standard Deviation, *OABSS *Over-Active Bladder Syndrome Score, *Duration *Disease duration, *H-Y* Hoehn and Yahr, *LEDD* Levodopa equivalent daily dose, *UPDRS *The Unified Parkinson’s Disease Rating Scale, *MMSE *Mini-Mental State Examination, *MoCA *Montreal Cognitive Assessment, *NMSQ *None Motor Symptom Questionnaire. OABSS ≤ 2 without OAB; 3–5 with mild OAB; 6–11 with moderate to severe OAB

### The OABSS score is not interacted with patients’ age, gender, LEDD, duration, or NMSQ in correlating with the risk of dyskinesia in PD patients

We conducted an analysis of the interaction effect to assess the combined influence of OABSS and patients’ age, gender, LEDD, duration and NMSQ on the occurrence of dyskinesia. The results of the analysis showed that OABSS did not exhibit any differential effects among subgroups of patients with different age (p for interaction = 0.545), gender (p for interaction = 0.872), LEDD (p for interaction = 0.134), duration (p for interaction = 0.715) and LEDD (p for interaction = 0.069) (Table 4). Hence, we can infer that the effect of OABSS on the occurrence of dyskinesia did not differ significantly between young and old age groups, male and female groups, and low and high LEDD, duration and NMSQ groups. Therefore, we conclude that the risk of dyskinesia significantly increases when moderate to severe OAB symptoms occur in PD patients.

**Table 4:** OABSS and Risk of dyskinesia, Stratified by Various Baseline Characteristics

	OABSS Interval			p for interaction
	≤2	3—5	6—15	
**Age > Median age (64)**				0.545
**Yes (** ***n =*** **628)**				
**Events/participants**	22/205	15/177	127/246	
**OR (95% CI)**	ref	0.770(0.380,1.524)^ns^	8.877(5.436,15.077)^***^	
**No (** ***n =*** **710)**				
**Events/participants**	29/322	11/163	94/225	
**OR (95% CI)**	ref	0.731(0.341,1.464)^ns^	7.250(4.609,11.701)^***^	
**Gender = male**				0.872
**Yes (** ***n =*** **788)**				
**Events/participants**	34/331	11/190	126/267	
**OR (95% CI)**	ref	0.537(0.254,1.054)^ns^	7.806(5.138,12.131)^***^	
**No (** ***n =*** **550)**				
**Events/participants**	17/196	15/150	95/204	
**OR (95% CI)**	ref	1.170(0.558,2.430)^ns^	9.177(5.320,16.680)^***^	
**LEDD > Median LEDD (337.5)**				0.134
**Yes (** ***n =*** **664)**				
**Events/participants**	39/215	15/164	161/285	
**OR (95% CI)**	ref	0.454(0.234,0.840)^*^	5.859(3.888,8.994)^***^	
**No (** ***n =*** **674)**				
**Events/participants**	12/312	11/176	60/186	
**OR (95% CI)**	ref	1.667(0.709,3.884)^ns^	11.905(6.404,23.932)^***^	
**Duration > Median Duration (36)**				0.715
**Yes (** ***n =*** **634)**				
**Events/participants**	35/201	15/147	166/286	
**OR (95% CI)**	ref	1.171(0.518,2.556) ^ns^	8.197(4.63,15.273) ^***^	
**No (** ***n =*** **704)**				
**Events/participants**	16/326	11/193	55/185	
**OR (95% CI)**	ref	0.539(0.275,1.011) ^ns^	6.561(4.294,10.241) ^***^	
**NMSQ > Median NMSQ (6)**				0.069
**Yes (** ***n =*** **590)**				
**Events/participants**	8/73	9/169	178/348	
**OR (95% CI)**	ref	1.055(0.570,1.874) ^ns^	5.137(3.162,8.373) ^***^	
**No (** ***n =*** **748)**				
**Events/participants**	43/454	17/171	43/123	
**OR (95% CI)**	ref	0.457(0.168,1.266) ^ns^	8.507(4.192,19.698) ^***^	

*Abbreviations*: *OABSS *Over-Active Bladder Syndrome Score, *LEDD *Levodopa equivalent daily dose, *NMSQ *None Motor Symptom Questionnaire, *ref *reference, *ns *not significant; *p < 0.05, ***p < 0.001

## Discussion

Parkinson’s disease (PD) is a chronic and progressive neurodegenerative disorder affecting dopaminergic neurons in the substantia nigra pars compacta of the brain [22]. Levodopa is the most common medication used to treat PD, which converts to dopamine in the brain, substituting for the lost neurotransmitter. However, long-term exposure to levodopa can lead to levodopa-induced dyskinesia [23–25], characterized by involuntary and abnormal movements of the limbs, face, and trunk. Research indicates that dyskinesia may be a result of pulsatile stimulation of dopamine receptors, which leads to the sensitization and heightened responsiveness of these receptors. Dyskinesia can have a detrimental effect on the quality of life for both patients and their caregivers [26]. When dyskinesia manifests, treating it effectively can prove to be a formidable challenge, even for experienced movement disorder specialists.

Given the formidable challenges associated with mitigating dyskinesia in patients with PD and its profound impact on their quality of life, it becomes imperative to devise proactive strategies for dyskinesia prevention and identify its risk factors. The intricate mechanism underlying dyskinesia in PD patients still eludes precise comprehension. Multiple risk factors have been implicated in the development of dyskinesia, including age at PD onset, gender (with a predilection for females), disease duration, sleep disorders, and the dosage and duration of L-DOPA medication exposure [5, 10, 24, 27]. In our present investigation, we corroborated these findings, reaffirming the significant association between dyskinesia and disease duration, LEDD, UPDRS II and MoCA scores. This conveys that dyskinesia arises not solely from medication usage but is intricately intertwined with disease severity and cognitive functioning [28]. In addition to these well-established insights, our study unearthed a compelling revelation—a notable surge in the incidence of moderate to severe OAB symptoms, encompassing urinary frequency, urgency, and nocturia, among PD patients grappling with dyskinesia. This indicates that moderate to severe OAB clinical symptoms are likely to suggest the likelihood of dyskinesia occurring.

The interplay between OAB and dyskinesia in PD is intricate and not yet fully elucidated. Empirical evidence demonstrates that deep brain stimulation can alleviate OAB severity in tandem with the UPDRS IV (dyskinesia) score in PD patients [29]. Previous case reports also identified a significant association between the use of the β3 adrenergic receptor agonist mirabegron, a medication for treating OAB symptoms, and the occurrence of severe Parkinsonian dyskinesia in a 72-year-old female patient, indicating the disturbance of acetylcholine, serotonin or noradrenaline release constructed a complex network that controls the occurrence of dyskinesia in individuals with PD [30]. Simultaneously, the emergence of dyskinesia could also be linked to disruptions in the basal ganglia nuclei, which possess an inhibitory effect on the voiding reflex [11, 31, 32]. In addition to the basal ganglia region, disturbances in the prefrontal cortical motor cortex, striatum, thalamic nucleus basalis, and cerebellar nuclei are all implicated in the development of dyskinesia [7]. Functional imaging reveals that voiding relates to cortical and subcortical structures such as the periaqueductal gray matter, basal ganglia, thalamus, insular cortex, anterior cingulate cortex, amygdala, and prefrontal cortex [33].

While the close relationship between dyskinesia and OAB was indicated, no previous research directly showed the evidence on their association specifically in PD patients. In the present study, the logistic regression on 1338 PD patients showed that disease duration, LEDD, urinary frequency, urgency, and nocturia, were independent risk factors for dyskinesia. In our logistic regression analysis, it was found that three OAB symptoms each served as independent risk factors for dyskinesia. This evidence has suggested the possibility of shared underlying mechanisms between OAB symptoms and dyskinesia in PD patients, particularly those with advanced stages. However, the precise etiology of OAB symptoms in PD patients remains elusive. Moreover, the impact of levodopa on OAB symptoms has yielded conflicting results [34–36], these studies point to a potential dichotomous role of dopamine receptor D1 and D2 in bladder function, with D1 exerting inhibitory modulation and D2 stimulating effects. Consequently, the loss of dopaminergic neurons, the density of D1/D2 receptors, and their respective sensitivities may collectively influence the bladder function in PD patients. Given the pathogenesis of dyskinesia and our study’s findings, we posit that the strong association between dyskinesia and OAB in PD patients, particularly those with progressive disease, could be linked to the relatively excessive use of levodopa and an imbalance in D1/D2 receptor function. Moreover, as previously mentioned, factors such as β3 receptors, acetylcholine, serotonin, and noradrenaline release are also implicated in the regulation of dyskinesia [30]. The intricate interplay between OAB and dyskinesia is evidently not dictated by a singular signaling pathway, but rather evolves from a multifaceted regulatory network. As such, deeper exploration of this complex relationship is imperative for future investigations.

While our study revealed a close association between OAB and dyskinesia, it is important to note that our research primarily relied on retrospective analysis. Further confirmation of these findings requires large-scale, longitudinal, prospective studies. Additionally, our study was conducted at a single center, and future research should include multi-center studies to eliminate potential biases. Objective indicators to evaluate urinary system function, such as urodynamic testing, should also be applied to eliminate the influence of self-estimated questionnaire on patients’ authentic urination condition. In future research, we will further explore the influence of medications including M-receptor blockers, β3 receptor agonists, and α receptor blockers on dyskinesia, and conduct in vivo animal experiments to explore the common signaling pathways between OAB and dyskinesia, in order to find new treatment targets.

In conclusion, our study, which included a large cohort of PD patients, confirms the close relationship between OAB and dyskinesia in PD patients through comprehensive analysis. Further investigation into these conditions could uncover the underlying mechanisms behind the development of various symptoms in PD patients, thereby contributing to a deeper understanding of PD’s pathogenesis and progression. Ultimately, this knowledge could lead to the identification of potential therapeutic targets for PD treatment, enhancing the efficacy of clinical interventions.

## Data Availability

No datasets were generated or analysed during the current study.
